# Reduction of Motion Artifacts in Cardiorespiratory Vital Signs as Oxygen Saturation and Non-Invasive Blood Pressure Through Redundant Denoising and Adaptive Filtering Methods for Wearable Healthcare Monitoring Systems

**DOI:** 10.1007/s13239-026-00850-0

**Published:** 2026-08-03

**Authors:** Fabian Andres Castaño, Alher Mauricio Hernández

**Affiliations:** 1 Bioinstrumentation and Clinical Engineering Research Group - GIBIC, Bioengineering Department, Engineering Faculty, Universidad de Antioquia UdeA, Calle 70 No. 52-21, Medellín, 050010 Colombia; 2 Physics Institute,Exact and Natural Sciences Faculty, Universidad de Antioquia UdeA, Calle 70 No. 52-21, Medellín, 050010 Colombia

**Keywords:** Signal denoising, Motion artifacts, Biomedical signal processing, Photoplethysmography, Non-invasive blood pressure, Home health care

## Abstract

**Background and objective:**

Chronic cardiovascular diseases have motivated the development of wearable systems capable of continuously monitoring physiological variables such as oxygen saturation (SpO$_2$) and non-invasive blood pressure (NIBP). However, motion artifacts remain one of the main limitations affecting the reliability of these measurements in ambulatory environments. This article presents a novel framework for motion artifact reduction in photoplethysmography (PPG) and NIBP signals based on redundant sensing, inertial measurements, ECG-guided temporal synchrony, and multichannel signal processing.

**Methods:**

The proposed framework combines independent component analysis and recursive least-squares adaptive filtering to separate physiological information from motion-induced interference. Performance was compared with conventional finite impulse response (FIR) filtering and wavelet shrinkage (WS) methods using signal-to-noise ratio (SNR), weighted distortion assessment (WDA), oxygen saturation estimation, and blood pressure estimation metrics.

**Results:**

The proposed method achieved statistically significant improvements in signal quality and physiological parameter estimation. Average SNR improved from -5.93±5.05 dB to -3.43±2.02 dB in the red PPG channel (p=0.02), while the WDA index increased from 0.48 to 0.73. Oxygen saturation estimation improved from $80.87\pm 9.09\%$ in motion-contaminated signals to $94.16\pm 2.72\%$ after denoising (p=0.04), approaching resting measurements ($95.23\pm 2.31\%$). For NIBP signals, statistically significant improvements were also observed (p=0.045), particularly for systolic pressure estimation, although diastolic pressure remained affected by residual motion-related distortions.

**Conclusions:**

The proposed framework improves the robustness of wearable PPG and NIBP monitoring under motion conditions through the combined use of sensor redundancy, ECG-guided temporal coupling, inertial measurements, and adaptive multichannel signal processing. The results demonstrate significant improvements in signal quality and physiological parameter estimation compared with conventional denoising approaches, supporting the potential use of the method in ambulatory cardiovascular monitoring applications.

## Introduction

Chronic cardiovascular diseases remain a major global health concern, as they are the leading cause of premature mortality worldwide and accounted for a substantial proportion of the approximately 18 million deaths from noncommunicable diseases among individuals under 70 years of age in 2021 [1]. To reduce the burden associated with these diseases and improve long-term patient management, information and communication technologies have enabled telemedicine platforms that facilitate continuous remote interaction between physicians and patients [2, 3]. The effectiveness of such platforms depends on the availability of reliable physiological information acquired outside traditional clinical environments. Wearable monitoring devices provide this information by continuously measuring cardiovascular variables and transmitting them to telemedicine systems for remote assessment and decision support, thereby enabling continuous and personalized healthcare delivery [4]. Consequently, the demand for reliable out-of-hospital monitoring has driven the development of wearable devices capable of measuring cardiovascular variables such as Electrocardiographic (ECG), Photoplethysmographic (PPG), and Non-Invasive Blood Pressure (NIBP) in ambulatory settings [5, 6].

Despite considerable advances in wearable monitoring technologies, reliable acquisition of physiological signals remains challenging due to the presence of artifacts that distort the signals of interest [7–9]. Common artifacts, including Power Line Interference (PLI), Baseline Wander (BW), and Motion Artifacts (MA), exhibit nonlinear, non-stationary, and unpredictable characteristics that complicate their identification and removal. Among them, motion artifacts are particularly problematic in ambulatory monitoring because they can significantly alter signal morphology and compromise the estimation of clinically relevant physiological parameters. As a result, ensuring signal quality remains a major obstacle for the widespread adoption of wearable systems in home healthcare and telemedicine applications.

Because diagnostic information is largely contained in the morphology of physiological waveforms, preserving signal integrity during denoising is a critical requirement. Numerous signal processing approaches have been proposed for artifact reduction, including Wavelet Shrinkage (WS), Empirical Mode Decomposition (EMD), Independent Component Analysis (ICA), Wavelet Independent Component Analysis (WICA), Empirical Mode Decomposition-Independent Component Analysis (EICA), and Adaptive Filters (AF) [10]. Although these methods can reduce noise contamination, they may also distort waveform characteristics that are essential for subsequent physiological analysis. Consequently, alternative strategies based on improvements in the acquisition process have been investigated to provide additional information that facilitates the separation of physiological signals from artifacts [10, 11].

Recent studies on motion artifact reduction in wearable PPG systems have explored deep learning, multimodal fusion, and multichannel acquisition strategies [12–15]. Deep learning approaches have demonstrated promising denoising performance; however, they generally require large annotated datasets, offline training procedures, and substantial computational resources, which may limit their implementation in resource-constrained wearable devices. Similarly, multimodal and multichannel approaches improve robustness by exploiting redundant information from inertial or optical sensors, but many focus primarily on heart-rate estimation rather than preserving the complete physiological waveform required for broader cardiovascular assessment [16–19]. These limitations highlight the need for methods capable of reducing motion artifacts while preserving waveform morphology and maintaining real-time feasibility.

In biomedical signal processing, signal distortion refers to alterations in the natural waveform caused by external processes or disturbances that may lead to information loss. One strategy to mitigate this problem is redundant measurement, in which information from a physiological process is acquired simultaneously at multiple sensing locations. When combined with motion information obtained from Inertial Measurement Unit (IMUs), redundant acquisition can provide complementary information that facilitates artifact identification and suppression. Furthermore, simultaneous acquisition preserves the temporal relationships among physiological signals. In this work, this coordinated acquisition framework is referred to as multichannel synchrony.

This article presents two denoising methods for reducing motion artifacts in PPG and NIBP signals. Both methods exploit redundant measurements of the signals of interest together with simultaneous motion recordings acquired from Inertial Measurement Unit (IMUs). The proposed PPG denoising method employs ICA to separate physiological and artifact-related components, followed by an adaptive filtering strategy for oxygen saturation estimation [20]. The NIBP denoising method is based on redundant and simultaneous pressure measurements combined with adaptive algorithms [21]. In addition, both methods incorporate the temporal synchrony between ECG, PPG, and NIBP signals to identify physiologically consistent cardiac events. Although these signals represent different physiological phenomena, they are temporally coupled through the cardiovascular cycle and therefore exhibit meaningful physiological relationships [22]. The proposed framework exploits these relationships to improve artifact reduction while preserving waveform morphology. Finally, the performance of the proposed methods is evaluated against state-of-the-art approaches using quantitative metrics including Signal-to-Noise Ratio (SNR), waveform distortion analysis, oxygen saturation estimation, and non-invasive blood pressure calculation [10, 23].

## Materials and Methods

This work presents two methods for reducing motion artifacts in PPG and NIBP signals to enhance the measurement process of oxygen saturation and non-invasive blood pressure. These methods utilize redundant and simultaneous measurement of each signal, along with synchronization with the ECG signal and the movement signal acquired from Inertial Measurement Unit (IMUs). The protocol for registering the signals of interest, the theoretical background of the denoising methods employed, and the proposed artifact reduction methods are detailed. The proposed framework consists of two independent motion artifact reduction methods: (1) a redundant PPG denoising method for oxygen saturation estimation and (2) a redundant NIBP denoising method for blood pressure estimation. The following subsections describe each method in detail.

### Population Size Selection

To ensure the statistical validity of the results, the minimum required sample size was estimated based on a statistical power analysis. The calculation was performed using expected variability values for physiological measurements (oxygen saturation and blood pressure) in healthy individuals at rest, as reported in the literature [24].

The sample size was determined using the formula for proportion studies with a one-sided hypothesis test (Eq. 1), which is commonly employed in health research [24, 25]:

1 $$\begin{aligned} N=\left( \frac{\sqrt{\theta (1-\theta )}Z_\beta +0.5Z_\alpha }{0.5-\theta }\right) ^2 = 19.24 \approx 20, \end{aligned}$$

where *N* is the minimum required sample size, θ is the expected proportion under the alternative hypothesis (0.8, corresponding to an anticipated 80% effectiveness in signal quality improvement), $Z_{\alpha }$ is the critical value associated with a significance level of α=0.05 (two-tailed), corresponding to 1.96, and $Z_{\beta }$ is the critical value associated with a statistical power of (1-β)=0.80, corresponding to 0.84.

The sample size calculation indicated that a minimum of 20 subjects was required to achieve 80% statistical power at a significance level of 0.05. Consequently, the study population of 20 healthy volunteers satisfied the minimum sample size requirement established by the statistical analysis. This sample size is also consistent with previous studies in wearable cardiovascular monitoring, including Shafer et al. (n=12) [26], Lupo et al. (n=20) [27], and the PPG-DaLiA dataset (n=15) [28].

### Register Protocol

The Photoplethysmographic, Non-Invasive Blood Pressure, and movement signals were recorded from a population of 20 healthy volunteers aged between 26±6 with a BMI of 24±5. The recording protocol was carried out continuously for each volunteer during three activities: 1) rest before movement, 2) controlled movement in laboratory conditions, and 3) rest after movement. Each volunteer was instructed to perform each stage for 5 minutes while physiological variables of interest were measured. During the second stage, participants walked at a normal travel speed of 4.2±0.8 km/h for 5 minutes. The movements involved arm swinging, wrist rotation, and posture changes, intentionally introducing inter- and intra-subject variability representative of typical wearable use scenarios. The motion protocol was designed to reproduce common movements encountered during ambulatory wearable monitoring, including walking, arm swinging, wrist rotation, and posture transitions, which are known sources of motion artifacts in PPG and NIBP recordings. The Ethics Committee for Human Studies of the Universidad de Antioquia approved the recording protocol (Approval 16–59-711).

**Fig. 1:**
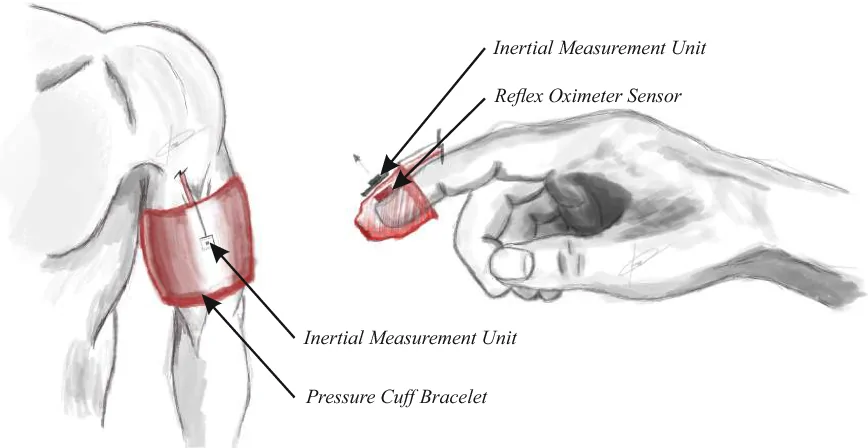
Location of pressure and photoplethysmographic sensors for redundant and simultaneous acquisition of signals

The acquisition of the red and infrared PPG signals was performed by a pair of reflective PPG sensors each located on the index fingers of each hand of the volunteers [29], pressure signals were acquired through the oscillometric method in which use an occlusion cuff to detect the pressure made by the blood on the walls of the brachial artery in the volunteer’s arm [30]. In this case, a bracelet located on each volunteer’s arm was used to obtain the signals so redundant and simultaneous. Additionally, the acquisition of an ECG derivation was performed to evaluate the multichannel synchrony between the physiological signals. These sensors have modifications in the acquisition hardware to integrate Inertial Measurement Unit (IMUs) into each sensor and acquire the movement pattern. Figure 1 shows the location of the sensors on the volunteer. In these, it is observed that both the bracelet and the reflective sensor have an IMU to movement measurement.

All reported values are expressed as mean ± standard deviation and were computed across n=20 independent datasets, each corresponding to one healthy volunteer. For signal processing purposes, each recording (5 min in duration) was segmented into frames of 2048 samples, yielding a total of 360 frames; however, statistical analysis was performed at the subject level.

### Signal Acquisition and Preprocessing

Electrocardiogram (ECG), photoplethysmography (PPG), non-invasive blood pressure (NIBP), and inertial measurement unit (IMU) signals were acquired simultaneously using a synchronized multichannel acquisition system. ECG signals were sampled at 250 Hz with a resolution of 24 bits. PPG and NIBP signals were sampled at 125 Hz with 12-bit resolution, while IMU signals were sampled at 25 Hz with a resolution of 16 bits.

All signals were temporally synchronized at acquisition to ensure accurate inter-signal alignment, which is critical for multichannel adaptive filtering and artifact separation [31, 32]. Prior to processing, all signals were normalized to zero mean and unit variance in order to reduce scale-related bias and improve numerical stability of the adaptive algorithms.

No synthetic motion artifacts were simulated in this study. Instead, the proposed method was evaluated exclusively on real physiological signals acquired under natural motion conditions. Motion artifacts were characterized and inferred directly from the IMU measurements, which served as a reference for motion-related disturbances affecting the PPG and NIBP signals.

A single-lead ECG was acquired as a timing reference and sampled at 250 Hz. PPG, NIBP, and IMU signals were recorded simultaneously using a common acquisition clock, ensuring temporal synchronization across all modalities. ECG R-peaks were used to verify multichannel synchrony. Details of the acquisition hardware are provided in [31, 32].

#### Independent Component Analysis

Through the use of a signal mixing model, the ICA method allows the separation of different measured signals from different sources considered independent [33, 34]. It is necessary to have a set of observations $\textbf{x}=\left( x_1, x_2, \cdots , x_m\right) ^{T}$ taken from *m* sensors, each of these observations can be modeled as the linear combination of a set of signals $\textbf{s}=\left( s_1, s_2, \cdots , s_n\right) ^{T}$ [33, 35]. The mixing model is described from a mixing matrix $\textbf{A}=\left( \mathbf {a_1}, \mathbf {a_2}, \cdots , \mathbf {a_n}\right)$ compose by *n* vectors of mixture $\mathbf {a_i}$ with size m×1.

It is necessary to consider some assumptions as: 1) signals from sources must be independent; 2) components must have a non-Gaussian distribution, or just one component has a Gaussian distribution at most; 3) the mixture matrix $\textbf{A}$ must be a square matrix, which means that the number of sensors must be equal to the number of independent sources (m=n) [35]. The separation matrix $\textbf{W}=\left( \mathbf {w_{ij}}\right) _{n\times n}$, and the *n* separate signals $\textbf{Y}=\left( \mathbf {y_1}, \mathbf {y_2}, \cdots , \mathbf {y_n}\right) ^{T}$ can be obtained through the model presented in equation 2.

2 $$\begin{aligned} \textbf{Y}=\textbf{Wx}=\textbf{WAs}=\textbf{Gs} \end{aligned}$$

Where $\textbf{G}$ is the global matrix and ideally should be the identity matrix, but in real cases, some of the sources are noise source and should not be considered in the model to perform the denoising, some elements of the separation matrix $\textbf{W}$ must be forced to zero to reduce the influence of the artifacts over the interest signal [35].

Independent Component Analysis (ICA) was employed to separate motion-related and physiological components from the multichannel signals. The FastICA algorithm was used with a fixed-point iteration scheme. Prior to ICA processing, signals were segmented into windows of 2048 samples with no overlap between consecutive windows to ensure quasi-stationarity based on previous studies [10, 31].

The number of independent components was set equal to the number of input channels. Convergence was determined based on the change in the weight vectors between iterations, with a stopping criterion defined as a relative change below $10^{-6}$ or a maximum of 10000 iterations. Parameter values were determined through pilot experiments using a subset of recordings and were subsequently fixed for the entire evaluation to avoid parameter tuning on the test data.

#### Adaptive Filters Recursive Least Square

Adaptive filters are based on the use of error estimation and cancellation algorithms such as the normalized ecursive Least Square (RLS) error cancellation algorithm, where the signal that you want to filter $x\left( n\right)$ is used as the main input, which is composed of the signal of interest and the signal of MA. In addition, it requires a reference signal or desired signal that contains the information that you want to obtain $d\left( n\right)$. However, the filter architecture has allowed this signal to be taken from the external noise that contaminates the signal of interest, thus achieving that the output signal $y\left( n\right)$ contains only information from the contaminating MA, and the error signal $e\left( n\right)$ corresponds to the signal of interest [36, 37].

The adaptive filter structure contains a vector of coefficients $\textbf{w}=w_i \left( n\right)$ that correspond to the impulse response of a conventional Finite Impulse Response (FIR) filter, where *i* corresponds to the number of coefficients that compose the filter. These coefficients are adaptively adjusted using the RLS algorithm to align the filter response with the desired input $d\left( n\right)$ [38]. Equation 3 models the operation of the Adaptive Filters for the reduction of artifacts in the signal.

3 $$\begin{aligned} e(n)=d(n)-y(n)=d(n)-\sum _{k=0}^{n} {w_k (n) x(n-k)} \end{aligned}$$

In this study, the RLS adaptive filter was implemented following the formulation proposed by Haykin 2002. The filter order was set to 32 coefficients, with a forgetting factor of λ=0.95 and an initialization parameter of δ=1.0. The coefficient vector was initialized to zero and the inverse correlation matrix as $\textbf{P}_0=\delta \textbf{I}$. Synchronized IMU measurements were used as the reference input to model motion-related disturbances affecting the PPG and NIBP signals. These parameters were selected based on preliminary experiments and maintained unchanged for all subjects and recordings to ensure consistency and reproducibility.

### Redundant PPG and NIBP Signals Denoising Methods

The methods proposed in this work are based on the simultaneous and redundant acquisition of the PPG signal, the NIBP signal, the movement of the person and an ECG derivation. The separation of multichannel components is the basis of these methods to improve the signal.

**Fig. 2:**
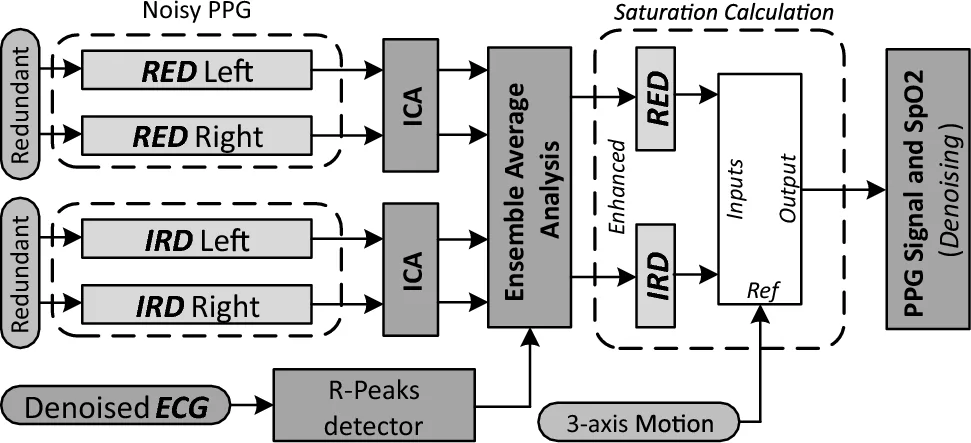
Block diagram representing the proposed denoising method to PPG signals. The method considers the simultaneous and redundant acquisition of the PPG signal contaminated with MA, the acquisition of a reference ECG signal, and motion signals

The method for artifact reduction in PPG signals focuses on the comparison and synchronization of PPG and ECG signals, exploiting the temporal coupling between cardiac electrical activation and peripheral pulse wave propagation throughout the cardiovascular cycle. The processing scheme of the proposed method is presented in Fig. 2.

**Fig. 3:**
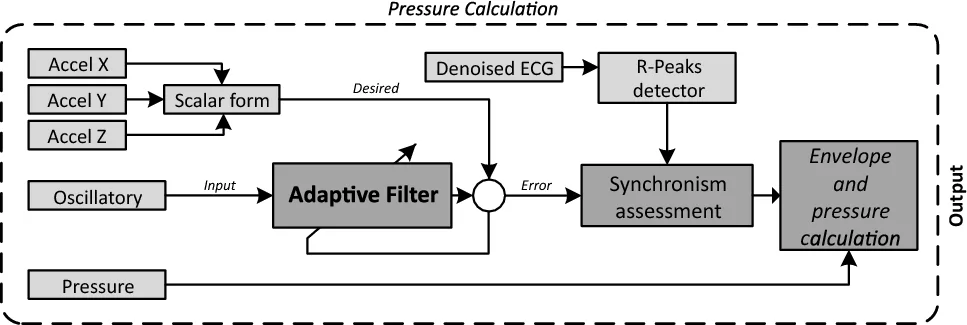
Block diagram representing the proposed denoising method to NIBP signals. The method considers the simultaneous and redundant acquisition of the NIBP signal contaminated with MA, the acquisition of a reference ECG signal, and motion signals

On the other hand, the method for reducing artifacts and calculating systolic, diastolic, and mean arterial pressure is founded on reducing MA using adaptive filtering (AF), with a reference signal acquired from Inertial Measurement Unit (IMUs). Once the enhanced signal is obtained, it seeks synchrony between the heartbeats represented in the ECG signal by the R peaks and in the NIBP signal by the maximum local peaks in the oscillatory signal. Figure 3 illustrates the proposed processing scheme for artifact reduction in the NIBP signal.

#### ECG Signal Denoising

To obtain the heartbeat synchronization base signal in the redundant signals, a derivation of the ECG signal was taken simultaneously with the PPG and NIBP signals, this ECG derivation was acquired under conditions of movement, so it was contaminated by artifacts. To perform the denoising of the signal, the Redundant denoising Independent Component Analysis (Rd-ICA) method was used, which takes the redundant information of the ECG signal and through the multichannel processing of the signal, the artifact component was separated from the interest signal [31, 39].

On the denoised ECG signal the R peaks were used to evaluate the synchrony with the PPG and NIBP signals, this evaluation allows to identify oscillations that not belong to the physiological process and that were introduced by the movement, considering that between two consecutive R peaks should to exist a single characteristic waveform of the PPG or NIBP signal as they correspond to a single heart beat [40]. In the case of the NIBP signal, this evaluation allowed to determine how useful the signal acquired during movement is to obtain systolic, diastolic and mean blood pressure information.

#### Multichannel Sources Separation

Considering that the interest information in the PPG signals comes from an independent source that is the blood flow through the capillaries when the heart makes a beat, and that the MA come from other different sources, the multichannel separation method through ICA was applied to the redundant acquired PPG signals, allowing to separate the MA component from the interest component, the method was applied separately to the red and infrared light signals.

Once the components of interest were obtained, the synchrony analysis was performed by comparing the waveform of the signal through the Ensemble Average (EA) method, allowing the identification of the red and infrared signals that were used to calculate the oxygen saturation.

#### Oxygen Saturation Calculation

**Fig. 4:**
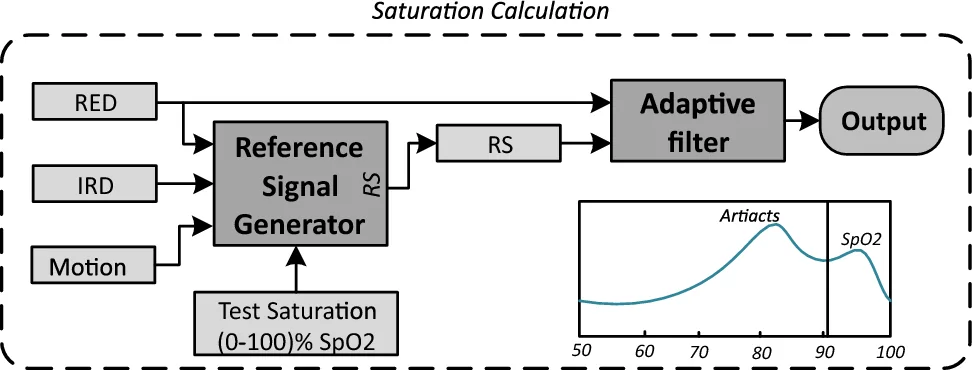
Block diagram of the proposed oxygen saturation estimation framework. Red and infrared PPG signals are acquired simultaneously with IMU-based motion measurements to generate reference and comparison signals for oxygen saturation estimation under motion conditions

To perform the oxygen saturation calculation, a modification to the algorithm known as Discrete Saturation Transform (DST) proposed by Masimo was made [20]. Figure 4 shows the calculation scheme of oxygen saturation.

In this, the red signal, the infrared signal and the movement signal are taken to generate a reference signal or desired signal. This reference signal $(\textbf{RS})$ contains information about the optical density ratio $(\textbf{r})$ which is unique for each oxygen saturation percentage value and is related through an empirical calibration equation [8, 41]. Equation 4 defines the reference signal.

4 $$\begin{aligned} \textbf{RS}=\textbf{r}'\times \textbf{IR} - \textbf{RD} + \textbf{M} \end{aligned}$$

Where $\textbf{RS}$ is the reference signal, $\textbf{r}$ is optical density ratio, $\textbf{IR}$ is infrared signal, $\textbf{RD}$ is red signal and $\textbf{M}$ is the motion signal. Finally, RMS value for the different values of $\textbf{r}$ is calculated at the output signal, where the maximum RMS value is found in the saturation zone corresponds to the percentage of oxygen saturation [20].

#### Scalar Form of Movement Vector

The movement information acquired by the inertial sensor is determined by three orthogonal signals defined by each of the Cartesian coordinate system directions (*x*, *y*, *z*). This vector can be transformed to its scalar form by calculating its magnitude and the average of its magnitude as shown in equation 5 [42].

5 $$\begin{aligned} a(t)=\text {mag}(\textbf{a}(t))-\text {mean}(\text {mag}(\textbf{a}(t))) \end{aligned}$$

Where *a*(*t*) represents scalar form of the vector, $\textbf{a}(t)$ is motion vector, $\text {mag}(\textbf{a}(t))$ refers to the motion vector magnitude and $\text {mean}(\text {mag}(\textbf{a}(t)))$ is the average of motion vector magnitude. This scalar form is used as the movement reference in the proposed denoising methods.

#### Calculation of Non-invasive Blood Pressure

Once the oscillatory signal of the NIBP signals is obtained, it is filtered through the adaptive filter in which it acts as an input *x*(*n*), the motion signal acts as the desired signal *d*(*n*), to get the output denoised *y*(*n*) as shown in Fig. 3. Once the denoised signal is obtained, the evaluation of the synchrony between the oscillatory signal and the ECG signal is performed, in which it is sought that between two consecutive R peaks only a characteristic undulation of the NIBP signal should appear. This allows to determine if the pressure measurement obtained from the signal can be masked by the MA [40]. When the denoised signal is obtained, the oscillometric method is applied in a conventional manner to calculate the systolic, diastolic and mean arterial pressures [43].

#### Signal-to-Noise Ratio

SNR has been used to calculate the improvement over the enhanced signal quantitatively and determine the performance of denoising techniques. The SNR improvement is calculated as the difference between the SNR of the output (enhanced signal) and input (reference signal) [44, 45]. This difference quantitatively shows the improvement in the signal in comparison with the contaminated signal. The SNR improvement is presented in equations 6, 7 and 8.

6 $$\begin{aligned} SNR_{in}\left[ {\,\mathrm{\text {dB}}}\right] =10\log \left( \frac{\sum _{i}{{\left| x_{c}\left( i\right) \right| }^2}}{\sum _{i}{{\left| x_{n}\left( i\right) -x_{c}\left( i\right) \right| }^2}}\right) \end{aligned}$$

7 $$\begin{aligned} SNR_{out}\left[ {\,\mathrm{\text {dB}}}\right] =10\log \left( \frac{\sum _{i}{{\left| x_{c}\left( i\right) \right| }^2}}{\sum _{i}{{\left| x_{d}\left( i\right) -x_{c}\left( i\right) \right| }^2}}\right) \end{aligned}$$

8 $$\begin{aligned} & SNR_{imp}\left[ {\,\mathrm{\text {dB}}}\right] =SNR_{out}-SNR_{in}\nonumber \\ & \quad =10\log \left( \frac{\sum _{i}{{\left| x_{n}\left( i\right) -x_{c}\left( i\right) \right| }^2}}{\sum _{i}{{\left| x_{d}\left( i\right) -x_{c}\left( i\right) \right| }^2}}\right) \end{aligned}$$

Where $x_c$ denotes the reference clean signal approximation, $x_n$ denotes the noisy signal, and $x_d$ represents the denoised signal.

In this study, the clean reference signal $x_c$ was approximated using the concurrently acquired redundant channel exhibiting the lowest motion contamination, as identified based on IMU-derived motion intensity metrics. Since an absolute artifact-free ground truth is not available during movement, this reference provides a practical surrogate for comparative SNR evaluation within the proposed redundancy-based acquisition framework. SNR values were computed over fixed-length 2048-sample non-overlapping signal segments and subsequently averaged across all segments and subjects.

#### Ensemble Average Method

The Ensemble Average method allows the characterization of a signal with a characteristic waveform that is repeated periodically. This method has been used to evaluate the PPG signal distortion introduced by MA and allows to evaluate the performance of denoising methods quantitatively considering the waveform of the signal. This is done by finding the average pattern of a signal that has a periodically repeated waveform [10].

In the EA method computing process, it was necessary to select a fiducial point on the standard waveform, which was the reference in time to synchronize the signal waveforms, the maximum amplitude wave of PPG characteristic waveform was selected as fiducial point. The size in time or in samples that has the standard waveform was determined to perform the partition of the signal, the synchronization through the fiducial points and the averaging of the signals. This time was determined as the time elapsed between two consecutive fiducial points. The computation of the EA is performed using the algorithm specified by Castaño et. al. 2019 [10].

In this work, the method was used to measure the performance presented by the denoising methods in the state of the art and to measure the performance of the proposed method in PPG signal denoising. Additionally, this method is used to determine the usefulness of the denoised signals to calculate the oxygen saturation percentage.

The estimation accuracy was quantified as the absolute error between physiological parameters extracted from the denoised signal and reference values obtained from synchronized ECG measurements, defined as is shown in Eq. 9.

9 $$\begin{aligned} EA = \left| \theta _{\textrm{est}} - \theta _{\textrm{ref}} \right| , \end{aligned}$$

where θ denotes the physiological parameter of interest, such as heart rate, pressure pulse high, or oxygen saturation.

#### Weighted Distortion Assessment (WDA)

The Weighted Distortion Assessment (WDA) index was used to evaluate the distortion introduced into the signal by the application of denoising methods, which makes use of the percentage difference between the characteristics of the improved signal and the resting signal measured from the EA Method. The index considers the signal distortion when taking into account the vector $(\Delta \textbf{EA})$ of the percentage of similarity of the characteristics, and the vector $(\Delta \textbf{W})$ of weighted weights for the features, and contains the weights to define the relevance that each feature will have on the calculation of the WDA index. Equation 10 presents the WDA index calculation method.

10 $$\begin{aligned} WDA\left( \textbf{EA},\textbf{W}\right) = \frac{\Delta \textbf{EA}\cdot {\Delta \textbf{W}}^{T}}{\mathopen |\Delta \textbf{W}\mathclose |} \end{aligned}$$

The coefficients’ values of the weight vector $\Delta \textbf{W}$ must be chosen between 0 and 1, these represent the percentage of relevance given to the preservation of a certain feature in the WDA performance index assessment. In the same way, values in $\Delta \textbf{EA}$ must be between 0 and 1.

In this work, the feature vector $\Delta \textbf{EA}$ includes morphological and temporal characteristics extracted from the ensemble-averaged PPG waveform, including peak amplitude, rise time, pulse width, and area under the curve. The corresponding weight vector $\Delta \textbf{W}$ was empirically selected to emphasize features most relevant for physiological interpretation.

### Validation and Assessment of Methods

To perform the validation of the denoising methods proposed in this work, the SNR index and the WDA index were used to measure the distortion introduced by the MA and how these are reduced through the denoising method. On the other hand, to evaluate the performance of the denoising method for the NIBP signal, the comparison of the performance presented by filtering methods such as the conventional FIR filters, and the denoising through WS, with respect to the redundant measurement method proposed in this article was performed.

#### Statistical Analysis

Statistical significance of performance improvements was assessed using paired two-tailed t-tests, comparing metrics obtained from the corrupted and denoised signals for each subject. This within-subject analysis was selected due to the repeated-measures nature of the experimental design. A significance level of p<0.05 was considered statistically significant.

## Results

This section presents the results obtained from applying the proposed denoising methods for PPG and NIBP signals. Additionally, it includes a comparison of the performance of artifact reduction methods presented in the state of the art with the methods proposed in this article. The results of the performance evaluation using the SNR and the EA method are also provided.

### Signal Acquisition and Preprocessing

**Fig. 5:**
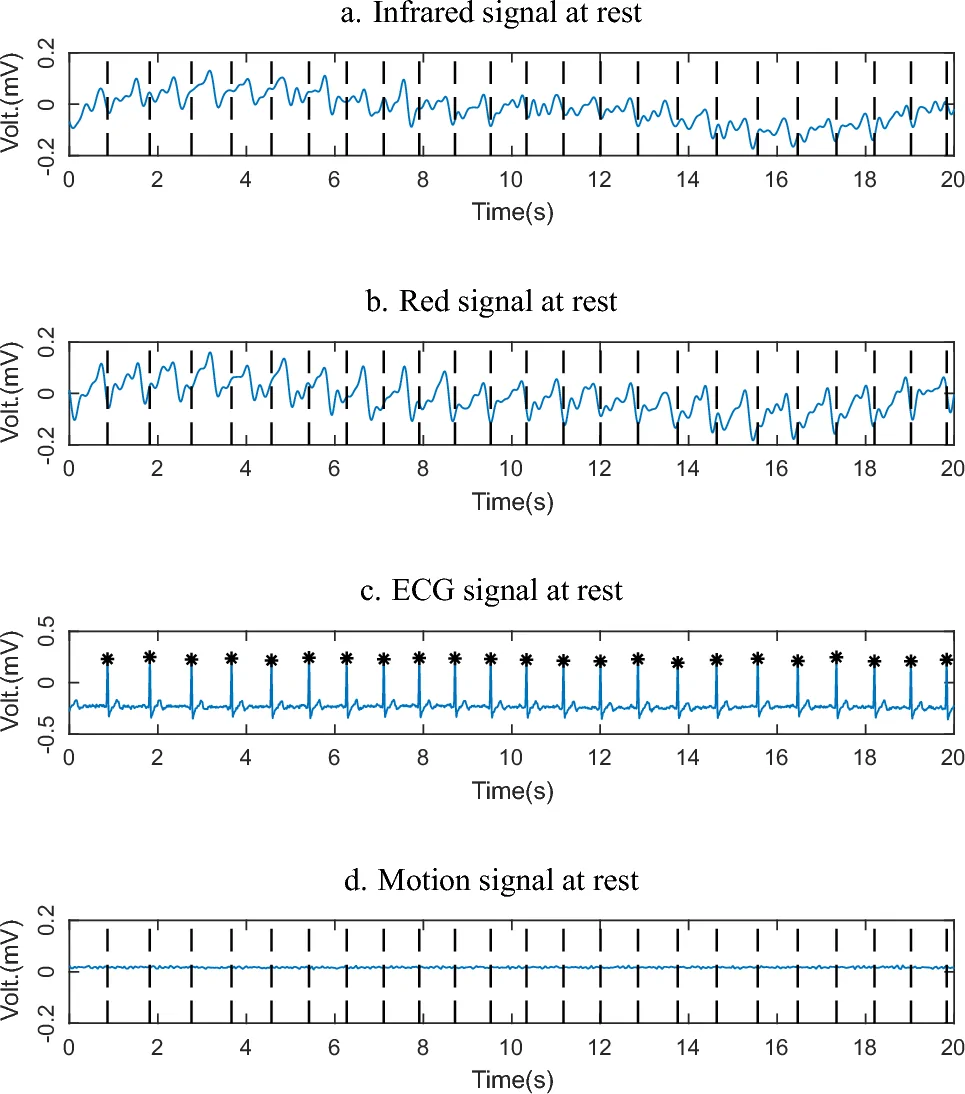
Epochs of 20 s of red and infrared Photoplethysmographic signals obtained in the left hand of a healthy volunteer at rest, in addition to the ECG lead II and motion signal of the PPG sensor. All signals were acquired simultaneously

As outlined in the register protocol section (section Register Protocol), simultaneous and redundant acquisition of PPG and NIBP signals was conducted on both arms of each volunteer. This acquisition took place while the volunteers were at rest and during controlled movement in a laboratory environment. Figure 5 illustrates the registration of a PPG signal obtained from a healthy volunteer at rest.

**Fig. 6:**
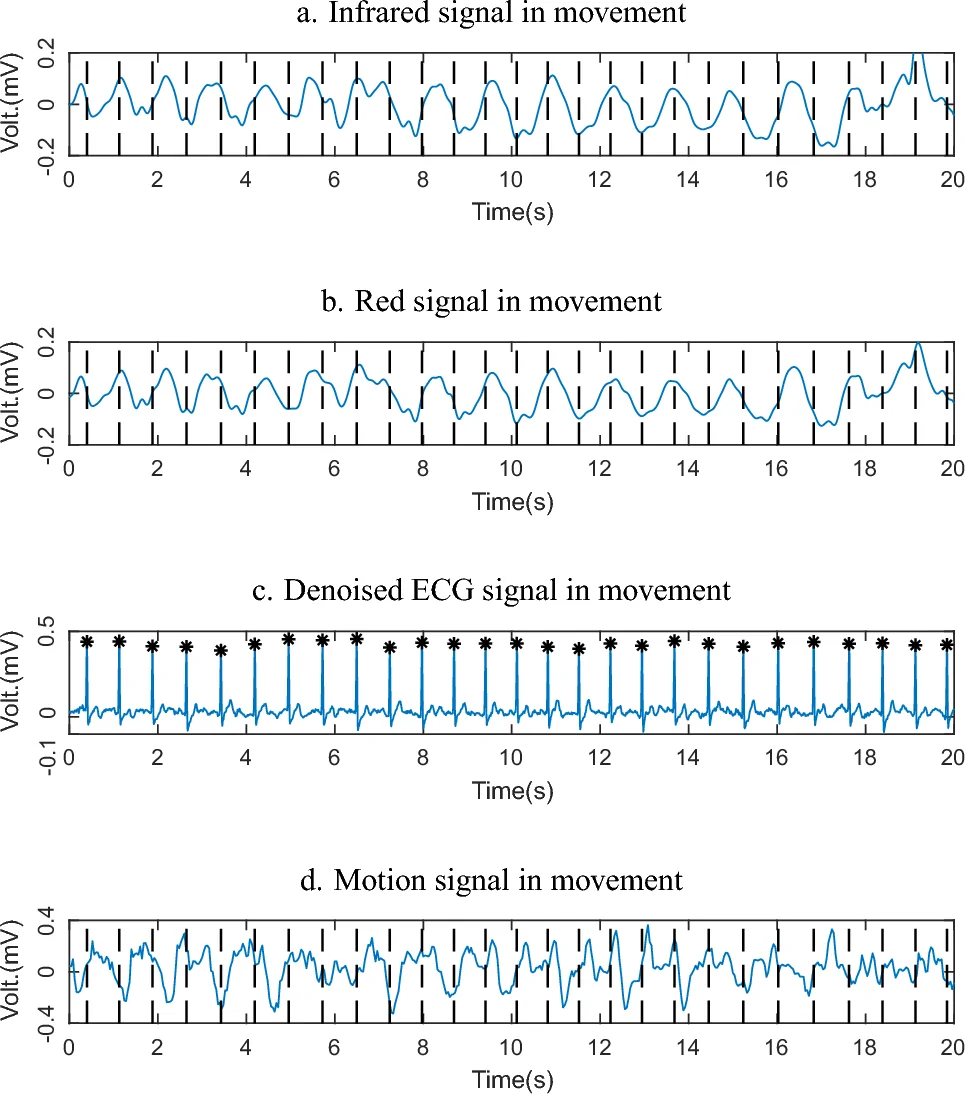
Epochs of 20 s of red and infrared Photoplethysmographic signals obtained in the left hand of a healthy volunteer while movement, in addition to the ECG lead II and motion signal of the PPG sensor. All signals were acquired simultaneously

Figure 5.a, b present the infrared and red PPG signals respectively, these were acquired at rest, the vertical lines refer to the temporal location of the R peaks of the ECG signal acquired simultaneously (Fig. 5c), giving evidence of the synchrony that exists between both signals that are physiologically related, this shows that for each characteristic waveform in the ECG signal only one characteristic waveform of the PPG signal is observed [22, 40]. Figure 5d shows the movement signal acquired from the Inertial Measurement Unit (IMUs). Figure 6 shows the registration of a PPG signal obtained from a healthy volunteer during the movement.

Similarly, Fig. 6a, b presents the PPG signal acquired in movement, Fig. 6c presents the ECG signal acquired during movement and improved through the Rd-ICA method. When evaluating the multichannel synchrony of both signals it was found that due to the MA there is no coincidence between the waveform of the ECG and PPG signals as evidenced in the signals acquired at rest. Figure 6d shows the movement of the PPG sensor.

To assess the relationship between motion and PPG signal corruption, the Pearson correlation coefficient was computed between the IMU acceleration magnitude and the PPG signal envelope during motion periods. A strong positive correlation between motion and PPG signal distortion was observed, as illustrated in Fig. 6a, b, and d. The correlation analysis yielded a statistically significant result (r>0.8, p<0.03), confirming that the IMU signal effectively captures motion components affecting the PPG signal.

**Fig. 7:**
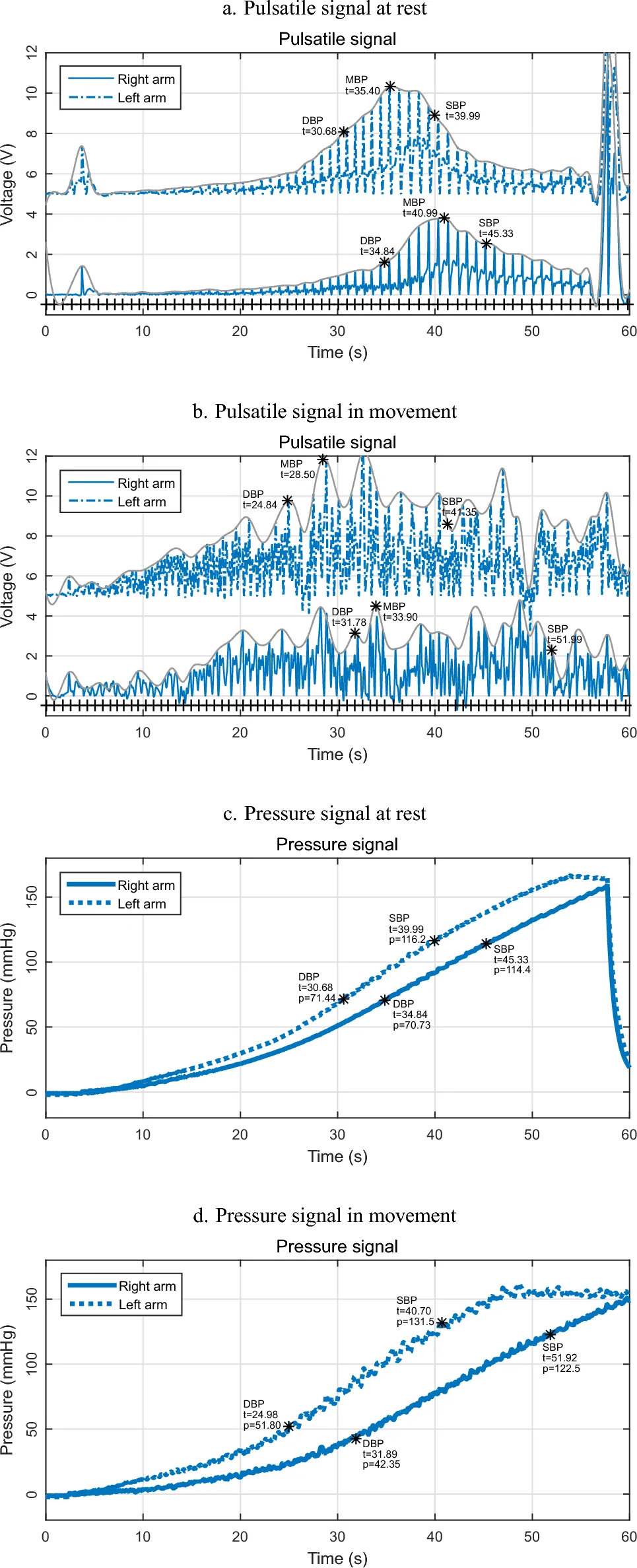
Oscillometric Non-Invasive Blood Pressure recordings obtained from a healthy volunteer under resting and motion conditions. Subfigures (**a**) and (**b**) show the pulsatile oscillation signals, while subfigures (c) and (d) present the corresponding cuff pressure signals. The annotated markers identify the measurement points used for systolic and diastolic blood pressure estimation based on oscillometric criteria

Figure 7 shows the non-invasive blood pressure signals acquired at rest and while movement respectively. Figures 7a, b show the pulsatile signal acquired by the cuff bracelets on both the left and right arms. Figures 7c, d show the pressure signal acquired in both arms.

In Figs. 7a,b, vertical lines are observed at the bottom, these lines correspond to the R peaks of the ECG signal that was acquired simultaneously, giving evidence of the multichannel synchrony between both signals, this synchrony is easily observed in the signals acquired at rest, while this synchrony is lost in contaminated signals due to the MA [22, 40].

### Redundant PPG and NIBP Signals Denoising Methods

**Fig. 8:**
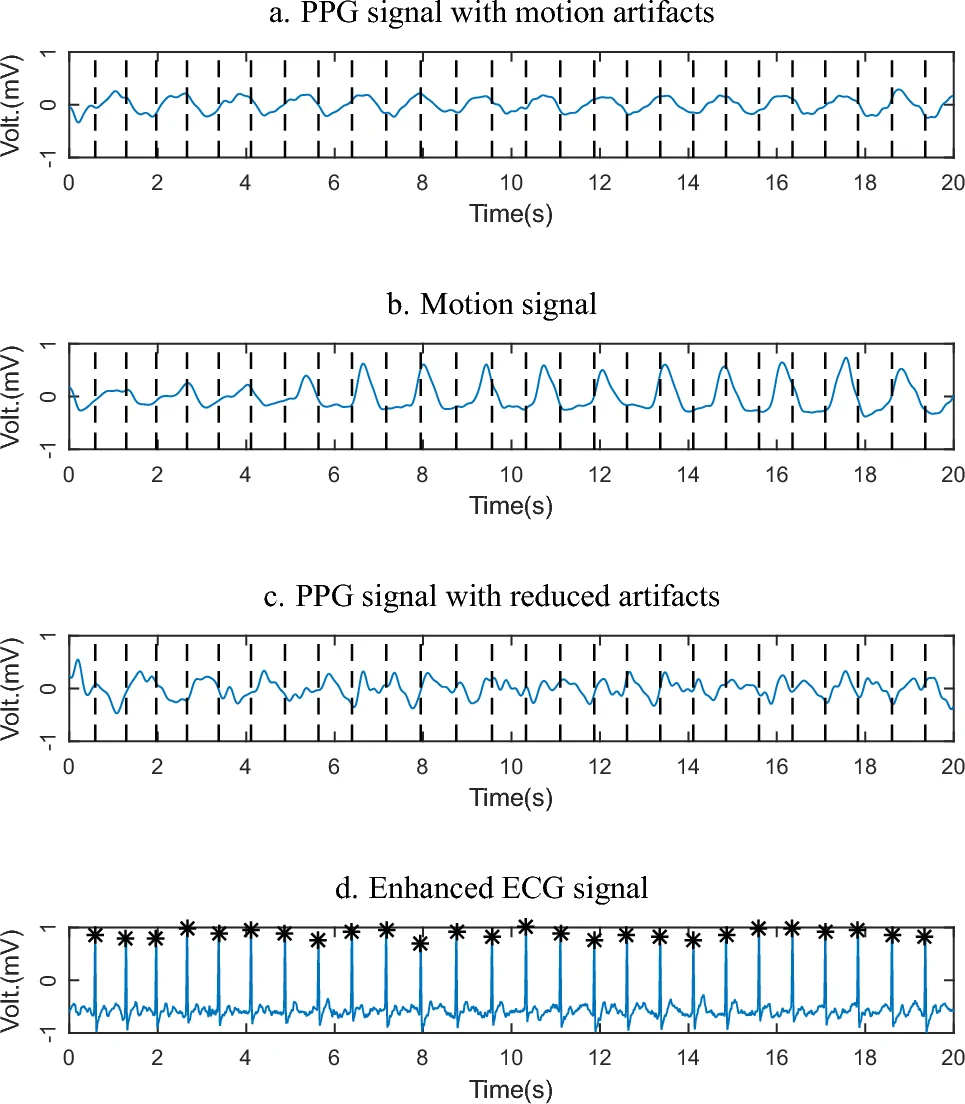
Epochs of 20 s extracted from Photoplethysmographic signals after application of the proposed motion artifact reduction method. Signal amplitude is shown in normalized units as a function of time. Vertical lines indicate synchronization with ECG R-peaks, illustrating heart rate coordination between PPG and ECG signals

The Redundant PPG denoising method was applied to the group of PPG signals to perform the reduction of MA from PPG signals acquired in both hands of the volunteer and the motion signal acquired from Inertial Measurement Unit (IMUs). Figure 8 presents the result of applying the proposed method for PPG signals.

Figure 8a presents the infrared light PPG signal contaminated by MA, Fig. 8b shows the movement signal acquired from the Inertial Measurement Unit (IMUs), Fig. 8c presents the improved signal from the MA reduction proposed method, and Fig. 8d shows the acquired ECG signal simultaneously and synchronized with the signals of interest, this synchrony is observed in the dashed vertical lines, which correspond to the moment when the R peak appears in the ECG signal.

### Non-invasive Blood Pressure Calculation

**Fig. 9:**
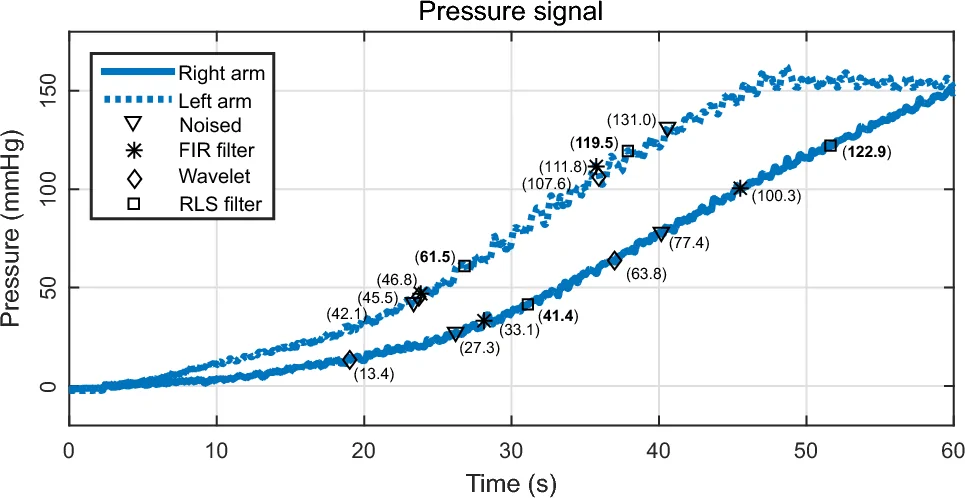
Pressure calculation from the pulsatile pressure signals using the different denoising methods presented in the state of the art and the method proposed in this article

To evaluate the performance of the proposed denoising method for the reduction of MA in pressure signals, some techniques in the state of the art such as conventional FIR filters and the WS were tested. Figure 9 presents the calculation result of systolic and diastolic blood pressure from the signals of each arm when applying the denoising methods. In bold, the result obtained from of the method proposed in this article is presented.

Table 1 shows the result of systolic and diastolic arterial pressures through the different methods of denoising from the signals of each arm, at rest and during movement. The denoising process also led to a statistically significant improvement in non-invasive blood pressure estimation under motion conditions (paired t-test, p=0.045). The improvement was particularly evident for systolic pressure, whose estimated values approached the physiological range observed during rest, whereas diastolic pressure remained more affected by residual motion-related distortions and therefore requires further methodological refinement.

**Table 1:** Result of the systolic and diastolic blood pressure after applying different denoising methods to NIBP signals

Pressure	At rest	MA	FIR Filter	WS	AF
Systolic	115.3±0.9	104.2±26.8	106.1±5.8	85.7±21.9	$\mathbf {121.2\pm 1.7}$
Diastolic	71.1±0.4	36.4±9.1	39.9±6.9	29.5±16.1	$\mathbf {51.5\pm 10.1}$

The values presented are found in $\left[ {\,\mathrm{\text {mmHg}}}\right] .$

Bold values indicate the best performance in the pressure calculation compared with rest measurement

### Ensemble Average Method and Saturation Calculation

**Fig. 10:**
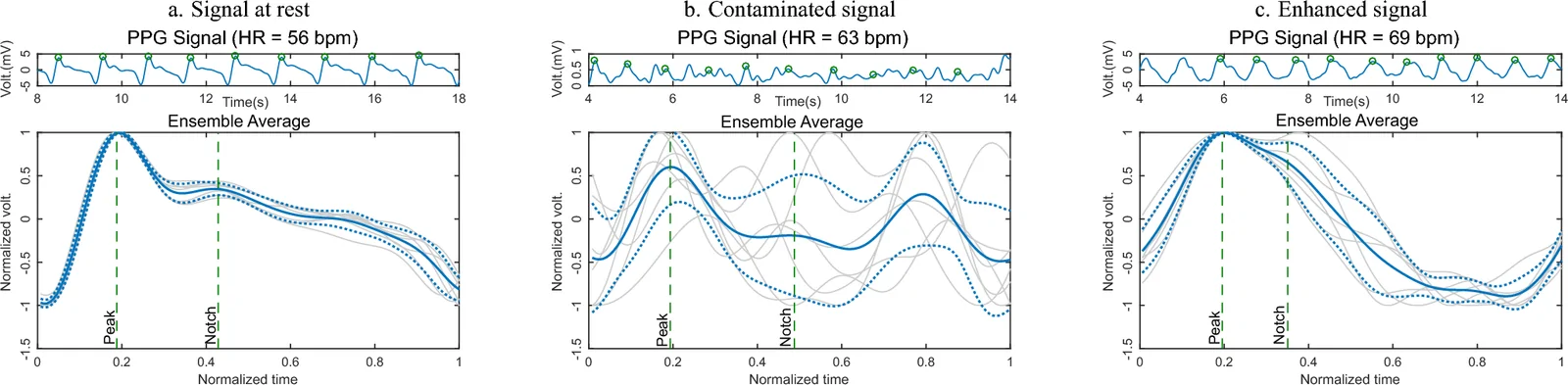
Ensemble Average method representation of PPG signals. The solid line corresponds to the ensemble-averaged waveform, while dashed lines represent ±1 standard deviation. Time is expressed in seconds and signal amplitude is shown in normalized units. Subfigures show (**a**) resting signal, (**b**) motion-contaminated signal, and (c) enhanced signal after denoising

**Fig. 11:**
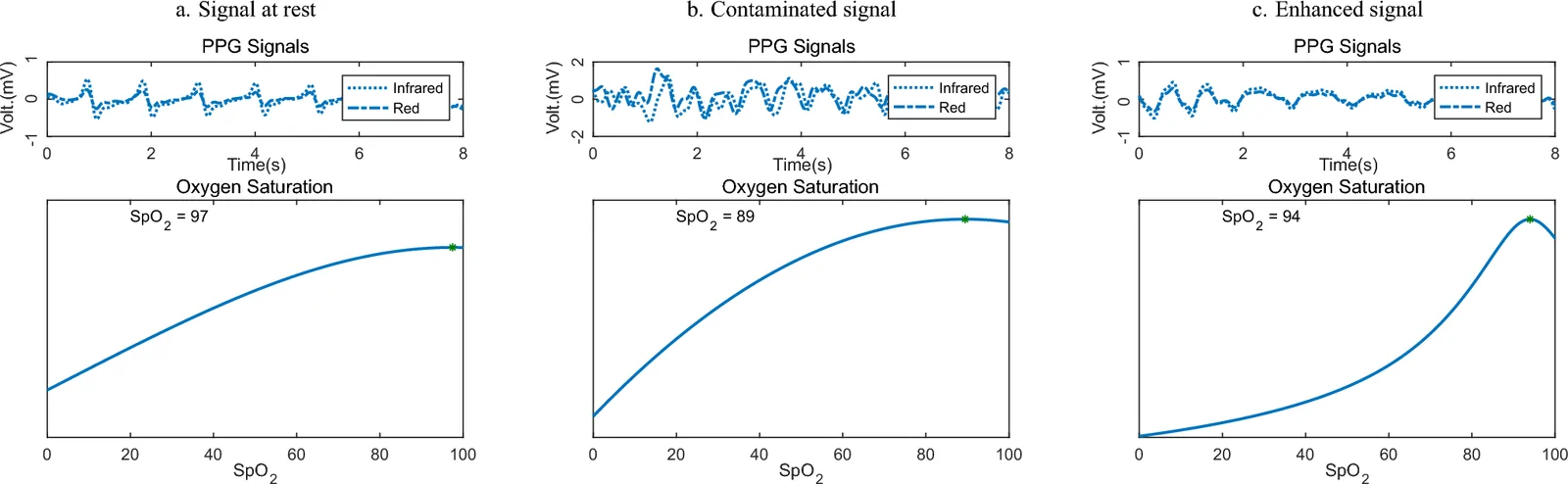
Calculation of oxygen saturation using the Discrete Saturation Transform method from Photoplethysmographic signals. Time is expressed in seconds and oxygen saturation in percentage (%). Subfigures show **a** resting condition, **b** motion-contaminated signal affected by Motion Artifacts, and** c** enhanced signal after application of the proposed denoising method

The Ensemble Average method was applied to evaluate the distortion in the PPG signal before and after the reduction of Motion Artifacts. The result of applying the EA method to PPG signals is presented in Fig. 10. After this, the calculation of the oxygen saturation from the PPG signals was performed, Fig. 11 presents the result of the calculation of the oxygen saturation.

In Fig. 10, solid line represents the average signal and the dashed lines represent the standard deviation signals. Figures 10.b and 10.c present the acquired signals in movement conditions and denoised signal, respectively; in the same way it presents the average signal by means of the continuous line and the standard deviations by means of the dashed lines. Bigger distortion of different waves that compose the EA is observed during the movement.

On the other hand, Fig. 11 shows the result of applying the Discrete Saturation Transform method on the PPG signals, a masking of the characteristics of the PPG signal is observed in Fig. 11.b leading to an error in the estimation of oxygen saturation.

### Oxygen Saturation Calculation

Table 2 presents the result of the oxygen saturation through the method of Discrete Saturation Transform for signals at rest before and after movement, signals contaminated with MA and signals improved from the proposed denoising method. A statistically significant improvement in oxygen saturation estimation was observed when comparing motion-contaminated and enhanced signals (paired t-test, p=0.04), indicating effective recovery of physiologically meaningful information after artifact reduction.

**Table 2:** Oxygen saturation through DST method for signals contaminated with MA

	Rest before	Motion	Enhanced	Rest after
$\text {SpO}_2$ [%]	95.23±2.31	80.87±9.09	94.16±2.72	91.77±1.60

### Signal-to-Noise Ratio

The $SNR_{imp}$ was calculated through equation 8 as the difference between the $SNR_{in}$ measured in the signal contaminated with Motion Artifacts and the $SNR_{out}$ measured in the enhanced signal from the denoising method. Table 3 presents the result of the SNR evaluation.

**Table 3:** SNR values for signals contaminated with MA acquired from healthy volunteers and enhanced PPG signals through denoising methods

Movement	$SNR_{in}$	$SNR_{out}$	$SNR_{imp}$
**Infrared**	-4.29±4.33	-3.32±1.26	0.97±3.92
**Red**	-5.93±5.05	-3.43±2.02	2.50±4.62

The values presented are found in $\left[ {\,\mathrm{\text {dB}}}\right]$

Further improvement is observed in the red signals compared to the infrared signals, which may indicate that there are more sources of artifacts that alter the correct acquisition of the infrared PPG signal. The improvement in SNR obtained after applying the proposed denoising method was statistically significant according to a paired two-tailed t-test (p=0.02), confirming a consistent enhancement in signal quality across subjects.

### Weighted Distortion Assessment (WDA)

On the other hand, the WDA index assessment was performed for the case of all features have equal relevance level. Table 4 shows the result of the distortion analysis through the WDA index for signals contaminated with MA and enhanced through denoising methods.

**Table 4:** Evaluation of the WDA index from the enhanced PPG signals

WDA	Movement	Denoised
**Case 1**	0.48	0.73

According to the definition of the WDA index, the previous result shows an acceptable improvement of the PPG signal for the calculation of oxygen saturation, as it tries to recover some of the main characteristics found in the waveform of the signal.

Statistical significance of the observed improvements was assessed using paired statistical tests with a significance level of p<0.05. Improvements in SNR, oxygen saturation estimation, and NIBP-related parameter estimation were found to be statistically significant (p=0.02, p=0.04, and p=0.045, respectively). Compared with motion-contaminated signals, the proposed approach achieved statistically significant improvements in signal quality and physiological parameter estimation. For NIBP signals, the improvement was more pronounced for systolic pressure, while diastolic pressure estimation remained affected by residual motion-related distortions, indicating that additional refinement is required for complete blood pressure recovery under motion conditions.

Beyond qualitative improvements, the proposed method provides measurable quantitative gains over baseline denoising techniques. For the red PPG channel, an average SNR improvement of approximately 25% was achieved compared to motion-contaminated signals. Similarly, the WDA index increased from 0.48 to 0.73, corresponding to a relative improvement of over 50% in waveform preservation. In terms of physiological relevance, oxygen saturation estimation during motion conditions improved by approximately 15% relative to conventional filtering approaches, yielding values closer to resting measurements. These results demonstrate consistent quantitative advantages over classical signal processing baselines such as FIR filtering and wavelet-based denoising, while maintaining real-time feasibility.

## Discussion

This article presents a method for reducing Motion Artifacts) in Photoplethysmographic and Non-Invasive Blood Pressure signals based on redundant and simultaneous acquisition of the signals of interest, inertial movement measurements, comparison with a reference ECG signal, and multichannel signal separation and processing techniques. The proposed approach was evaluated on data acquired from healthy volunteers performing controlled movements representative of typical wearable use, including arm swinging, wrist rotation, controlled walking, and changes in posture, introducing substantial inter- and intra-subject variability in signal quality. Performance was assessed through comparison with state-of-the-art methods using quantitative metrics such as SNR, waveform distortion indices (WDA), and the Ensemble Average method [10]. Reproducibility was ensured by applying an identical acquisition and processing pipeline across all subjects and motion conditions, yielding consistent trends in signal quality and physiological parameter estimation. Although the study was conducted on healthy volunteers, the proposed method relies on motion-induced signal characteristics captured by the IMU rather than subject-specific physiological models, supporting its potential generalization to real-world wearable scenarios. Further validation in broader populations and under unconstrained daily-life conditions remains an important direction for future work.

As confirmed in the results of this article, modifications in biomedical signal acquisition hardware can significantly improve the Motion Artifacts reduction in Photoplethysmographic and Non-Invasive Blood Pressure signals supported by multichannel signal processing techniques, as evidenced in the state of the art, the problem cannot be addressed only from software [46]. Hardware modifications were added to redundantly measure both Photoplethysmographic and Non-Invasive Blood Pressure signals, and for the measurement of movement through Inertial Measurement Unit (IMUs) which allows determining the type of signal contamination [31, 32, 47].

Figures 5 and 7.a present the simultaneous and redundant acquisition of the physiological signals at rest, illustrating the temporal coupling between ECG, PPG, and NIBP signals throughout the cardiovascular cycle. Under resting conditions, each ECG R-wave is followed by a corresponding peripheral pulse waveform in the PPG and NIBP signals, reflecting the physiological relationship between cardiac electrical activation, arterial pressure propagation, and peripheral blood volume changes [48]. During motion (Figs. 6 and 7.b), this temporal relationship becomes obscured by motion artifacts, motivating the use of the proposed multichannel synchrony framework to recover physiologically consistent signal components.

The characteristic waveform of the PPG signal presents two characteristic lobes of the pulse signal corresponding to the maximum stress moment (peak) and the rest time (notch) [22, 40]. Despite this, evaluating the characteristic waveform of the signal contaminated with Motion Artifacts, the loss of the characteristic waveform was observed (Fig. 10.b), which makes it unsuitable for the calculation of the saturation of oxygen. On the other hand, in Fig. 10.c an enhancement was observed concerning the contaminated signal, in this one tries to recover the characteristic waveform of the signal. It is also observed that the movement significantly affects the calculation of the heart rate from the PPG signal, this is another characteristic of the signal that is preserved by the proposed denoising method.

Figure 11 presented the oxygen saturation for signals acquired at rest, contaminated with Motion Artifacts and enhanced from the proposed denoising method. As seen in 11.b, contamination by Motion Artifacts masks the characteristics of the Photoplethysmographic signal, which affects considerably the result of the oxygen saturation calculation as this result can lead to misdiagnosis, the denoising method recovers some of the characteristics of the Photoplethysmographic signal, thus reducing errors in the calculation of oxygen saturation as shown in Figs. 11.a and 11.c, This result can be clearly seen in Table 2.

The comparison of the Non-Invasive Blood Pressure results obtained using different denoising methods is presented in Figure 9. The methods reported in the state of the art exhibited considerable variability between measurements obtained from both arms. In contrast, the proposed Adaptive Filters-based approach produced more consistent estimates across both measurement sites, as summarized in Table 4. Although the proposed method substantially improved the estimation of blood pressure parameters under motion conditions, the recovery was more pronounced for systolic pressure than for diastolic pressure. Furthermore, because the resting and motion measurements were acquired during different physiological states, some variation in blood pressure is expected independently of motion artifacts. Nevertheless, residual errors observed in diastolic pressure estimation indicate that further refinement of the denoising strategy is required to achieve more accurate blood pressure recovery during physical activity.

After the denoising of the signals, the conservation and recovery of some main characteristics of the signal were observed (Fig. 10), these characteristics of the signal waveform that had been masked by the Motion Artifacts allowed the calculation of the oxygen saturation in the moving signals, the improvement of the signal becomes evident when analyzing the result of the SNR (Table 3) and the WDA indices (Table 4) [49], in addition to the result of the standard deviations of the signals obtained from the Ensemble Average Method (Fig. 10).

Rather than targeting clinical-grade accuracy, the primary objective of this work is to assess the ability of the proposed approach to preserve physiologically relevant signal characteristics under motion. Improvements in SNR, waveform morphology (WDA), and physiological parameter consistency (SpO$_{2}$ and NIBP trends) collectively indicate effective motion artifact suppression in realistic acquisition conditions [18, 50].

The computational load of the proposed approach is primarily determined by the adaptive filtering and source separation stages. In the experimental setup, data acquisition and storage were performed using dsPIC33 microcontrollers coordinated by a PIC32MZ device, while signal processing was conducted offline in MATLAB to facilitate algorithm development and analysis. However, the proposed framework does not rely on computationally intensive operations or data-driven training procedures. Both the ICA and RLS-based adaptive filtering stages exhibit linear or near-linear computational complexity with respect to the signal length and number of channels.

Lightweight and fixed-point implementations of these algorithms have been widely reported and are currently available for embedded platforms. As a result, the proposed method can be migrated to modern microcontroller architectures, such as ARM Cortex-M7-based systems, enabling real-time operation in wearable devices. The frame-based processing strategy adopted in this work further supports real-time feasibility by limiting memory requirements and processing latency.

The proposed approach relies on hardware redundancy through multiple PPG channels and inertial sensors, which inevitably increases system complexity compared to single-sensor solutions. However, this redundancy is intentionally leveraged to improve robustness against motion artifacts and information loss, particularly in dynamic conditions where purely algorithmic approaches may fail. From a practical perspective, the additional sensors introduce moderate increases in power consumption and hardware cost; nevertheless, modern low-power optical sensors and MEMS IMUs exhibit minimal impact on the overall energy budget of wearable devices. Furthermore, the architecture is scalable, as redundant channels can be selectively enabled or disabled depending on the application requirements, allowing a trade-off between robustness and resource consumption. Advances in system-on-chip integration and sensor miniaturization make the proposed framework compatible with compact wearable platforms, where multi-sensor fusion is increasingly adopted to enhance signal reliability in real-world conditions.

A limitation of this study is the absence of an absolute ground truth signal for PPG and NIBP measurements under motion conditions. In real-world wearable scenarios, motion artifacts are inherently coupled with physiological signals, making it impractical to obtain a perfectly clean reference during movement. Consequently, the proposed method was evaluated using indirect but physically meaningful references, including redundant sensor channels and IMU-derived motion information [19, 50]. In addition, the experimental validation was conducted exclusively on healthy volunteers. This choice was intentional, as the primary objective of this work was to assess the robustness of the proposed denoising framework under controlled physiological conditions, thereby isolating motion-induced artifacts from pathological variability. By restricting the study population, the influence of confounding factors such as abnormal vascular compliance or altered hemodynamics was minimized, enabling a focused evaluation of signal processing performance. While these factors may limit direct extrapolation to clinical populations, the proposed method is not inherently dependent on subject-specific physiological models and can be extended to pathological conditions. Future work will therefore address validation in broader and clinical cohorts, as well as under unconstrained daily-life conditions.

The present study focuses on evaluating the performance of the complete multichannel framework for motion artifact reduction in PPG and NIBP signals. The individual concepts of sensor redundancy, physiological synchrony, and adaptive filtering were selected based on previous investigations and integrated into a unified processing pipeline [10]. Although the proposed method demonstrated superior performance compared with FIR and wavelet-based approaches, future work will include broader benchmarking and dedicated ablation studies to quantify the individual contribution of each component in PPG and NIBP signal recovery.

Unlike recent deep learning-based approaches, the proposed method focuses on hardware-level redundancy combined with adaptive signal processing, enabling real-time implementation without the need for training data [51]. Although the individual components (multichannel acquisition, ICA, adaptive filtering) have been previously explored, their integration for simultaneous PPG and NIBP acquisition in a wearable context, together with quantitative evaluation of physiological metrics (SpO$_2$ and blood pressure), represents the main contribution of this work.

The results presented in this article about the use of redundant measurements and multichannel synchrony for the detection of pulses in Photoplethysmographic and Non-Invasive Blood Pressure signals are satisfactory. This article presents an innovative method for reducing motion artifacts in vital signs, such as oxygen saturation and non-invasive blood pressure, enhancing performance in information extraction. This may prove useful in generating alerts about changes in the cardiorespiratory system, even in everyday work-life situations. This will allow for future monitoring of the cardiovascular status of workers identified as having high cardiovascular risk. Although the proposed framework demonstrated significant improvements in signal quality and physiological parameter stability during motion, clinical-grade validation against independent reference devices was beyond the scope of the present study and remains an important direction for future research.

While the presented results are promising, the method requires refinement to enhance its performance and minimize the elimination of significant physiological information currently discarded due to contamination by motion artifacts.

## Conclusions

This work presented a multichannel framework for motion artifact reduction in PPG and NIBP signals based on redundant sensing, IMU-assisted motion characterization, physiological synchrony with the ECG signal, and adaptive signal processing techniques. The proposed architecture improves signal quality while preserving waveform morphology, enabling more reliable estimation of oxygen saturation and non-invasive blood pressure under motion conditions.

Experimental results demonstrated consistent improvements in signal-to-noise ratio, waveform distortion metrics, and physiological parameter estimation compared with representative state-of-the-art denoising approaches. These findings indicate that combining hardware redundancy with adaptive processing provides advantages over approaches that rely exclusively on software-based artifact suppression.

An important contribution of this work is the integration of redundant acquisition, multichannel synchrony, and adaptive filtering into a unified framework that does not require prior model training and is compatible with real-time embedded implementation under some modifications. This makes the proposed approach attractive for wearable cardiovascular monitoring systems operating in dynamic environments.

Future work will focus on validating the proposed framework in larger and more diverse populations, including patients with cardiovascular disorders, performing ablation studies to quantify the contribution of each processing stage, and conducting clinical-grade evaluations against independent reference devices under unconstrained daily-life conditions.

## Data Availability

The datasets generated and analyzed during the current study are available from the corresponding author upon reasonable request.
